# Frailty-Based Remote Monitoring in Older Adults With Heart Failure: Conceptual Framework for Adaptive Digital Health Strategies

**DOI:** 10.2196/88681

**Published:** 2026-05-20

**Authors:** Rémi Esser, Olivier Maurou, Marine Larbaneix, Alejandro Mondragon, Marlène Esteban, Christine Farges, Nicolas Pages, Sophie Nisse Durgeat, Marc Harboun

**Affiliations:** 1 Hôpital La Porte Verte, 6 avenue du Maréchal Franchet d'Esperey, Versailles, Île-de-France, France, +33 139639628; 2 NP Medical, Bordeaux, France

**Keywords:** digital health strategy, remote monitoring, frailty, aging population, heart failure, telemedicine workflow

## Abstract

Remote monitoring is increasingly used in heart failure care, but most programs rely on uniform models that insufficiently reflect the heterogeneity of older adults, particularly with respect to frailty, cognitive impairment, functional dependency, and caregiver availability. This viewpoint argues that frailty should be considered a central determinant of remote monitoring design in older adults with heart failure, rather than a secondary modifier of conventional digital health pathways. Drawing on evidence from heart failure telemonitoring, geriatric medicine, and real-world cardiogeriatric experience, we propose a frailty-adaptive framework structured around four clinical trajectories: robust, prefrail, frail, and palliative. Each trajectory is associated with distinct monitoring objectives, workflow adaptations, and response pathways. Robust patients may benefit primarily from optimization, self-management support, and guideline-directed therapy titration; prefrail patients from early detection of deterioration and functional decline; frail patients from proxy-supported reporting, nurse-led triage, and rapid-access cardiogeriatric reassessment; and palliative patients from simplified symptom-guided monitoring focused on comfort, hospitalization avoidance, and caregiver support. This framework reframes remote monitoring as a stratified clinical process rather than a purely technological intervention. By aligning digital strategies with frailty status, functional capacity, and care goals, frailty-adaptive remote monitoring may improve clinical relevance, promote digital health equity, and support more sustainable models of care for older adults with heart failure.

## Introduction

### The Misalignment Between Digital Health and Aging Realities

Remote monitoring has become an integral component of heart failure (HF) management, aiming to reduce rehospitalizations and improve chronic disease control. Early telemonitoring trials such as Tele-HF (Telemonitoring to Improve Heart Failure Outcomes), TIM-HF (Telemedical Interventional Monitoring in Heart Failure), and TIM-HF2 (Telemedical Interventional Management in Heart Failure 2) predominantly enrolled relatively younger and less frail populations, limiting the generalizability of their findings to very old adults with multimorbidity and geriatric syndromes. More recent large-scale real-world programs, such as TELESAT-HF, have demonstrated heterogeneous but clinically meaningful results, strongly influenced by program structure, patient selection, and response workflows [1-3].

Much of the evidence base and many telemonitoring workflows were initially developed and validated in populations that were younger and less frail. In parallel, several studies have shown that a substantial proportion of older adults experience “telemedicine unreadiness,” driven by cognitive impairment, sensory limitations, and reduced digital literacy [4-6]. Together, these observations suggest that existing telemonitoring models may not be fully aligned with the characteristics and needs of very old and frail populations, in whom HF is closely intertwined with multimorbidity, functional decline, and dependency.

Frailty is a well-established clinical construct reflecting reduced physiological reserve and increased vulnerability to stressors. Seminal work by Fried et al [7] and Rockwood et al [8] defined frailty as a measurable and clinically meaningful condition, later expanded by Clegg et al [9] as a major determinant of health outcomes at the population level. In HF, frailty has been consistently associated with higher risks of hospitalization, disability, and mortality, often outperforming traditional cardiac markers in prognostic accuracy among older adults [10-13]. At the same time, the global burden of HF continues to rise with population aging [14]. However, frailty remains largely absent from the design of most telemonitoring strategies, including patient selection, alert thresholds, and care pathways.

Although several studies have shown that older adults can successfully engage in telemonitoring when appropriately supported [4-6], adherence, comprehension, and responsiveness vary substantially according to frailty level.

This viewpoint argues that frailty should be considered a primary determinant of remote monitoring design in older adults with HF, rather than a secondary modifier. We propose a structured frailty-adaptive framework based on 4 major clinical trajectories—robust, prefrail, frail, and palliative—each associated with distinct monitoring objectives, clinical responses, and organizational requirements.

By reframing remote monitoring as a stratified, patient-centered strategy rather than a purely technological intervention, this approach aims to improve clinical relevance, optimize resource allocation, and promote digital health equity. Integrating frailty into telemonitoring design is essential to ensure that digital interventions remain accessible, effective, and safe across the full spectrum of aging and vulnerability.

### Conceptual Basis of the Framework

#### Conceptual Basis and Sources

This viewpoint is based on a narrative synthesis of the literature combined with real-world clinical experience from a cardiogeriatric telemonitoring program. To our knowledge, existing telemonitoring approaches do not explicitly integrate frailty into digital health planning for older adults with HF nor link segmentation to practical determinants such as proxy reporting, nurse-led triage, or rapid-access pathways.

Frailty can be assessed using validated tools such as the Fried phenotype or the Clinical Frailty Scale. The 4 profiles proposed in this framework correspond to clinically meaningful frailty trajectories rather than rigid categories, allowing flexibility based on clinical judgment and validated instruments.

#### Frailty Segmentation Approach

In this framework, frailty is used as an integrative construct reflecting global vulnerability, rather than being limited to digital capability or functional independence.

Frailty should not be interpreted solely as a proxy for digital literacy or autonomy. While some frail individuals may retain the ability to engage with digital tools, functional independence, cognitive status, and caregiver availability represent complementary dimensions that influence telemonitoring feasibility.

Previous studies have demonstrated that HF trajectories in older adults are highly heterogeneous and strongly influenced by frailty and functional status rather than left ventricular ejection fraction alone [9-13,15-17].

Within this perspective, remote monitoring is conceptualized not as a purely technological intervention but as a stratified care strategy, in which monitoring objectives, intensity, and response pathways are adapted to frailty level.

#### Illustrative Real-World Telemonitoring Program

To support the proposed framework, we describe an illustrative cardiogeriatric telemonitoring program reflecting real-world clinical practice.

This large real-world cardiogeriatric telemonitoring program involved approximately 568 older adults with HF over a median monitoring duration of 18 months within a care pathway that integrates telemonitoring, nurse-led triage, and cardiogeriatric day-hospital reassessment. These figures are provided to contextualize the clinical experience underlying this viewpoint and should not be interpreted as original outcome data.

Patient follow-up relied on a structured symptom-based questionnaire completed twice weekly, focusing on dyspnea, peripheral congestion, weight evolution, fatigue, and other indicators of HF decompensation. When patients were unable to complete the questionnaire themselves—frequent among frail or cognitively impaired individuals—data were entered by family caregivers, home-care nurses, or residential-care staff.

Responses were processed through an automated algorithm generating 3 levels of alerts (green, orange, red) based on predefined clinical thresholds. Alerts were transmitted to a specialized cardiogeriatric nursing team, who performed structured telephone assessments to confirm symptoms and evaluate clinical status.

When indicated, interventions included adjustment of oral diuretic therapy according to predefined protocols or rapid referral to a cardiogeriatric day-hospital pathway.

This workflow illustrates a model in which remote monitoring is supported by algorithm-based alert systems, nurse-led triage, and rapid access to in-person reassessment. The program described here is provided as an illustrative organizational model, rather than a device-specific intervention. The underlying principles are consistent with telemonitoring pathways implemented in France within the ETAPES (Expérimentations de Télémédecine pour l’Amélioration des Parcours en Santé) program and are broadly applicable across different digital health platforms.

The program described here is provided as an illustrative organizational model, rather than a device-specific intervention. The underlying principles are consistent with telemonitoring pathways implemented in France within the ETAPES program and are broadly applicable across different digital-health platforms.

#### Adaptation to Functional and Digital Limitations

An essential component of this framework is the adaptation of telemonitoring to differences in digital literacy, sensory capacity, and functional autonomy among older adults.

In real-world practice, a substantial proportion of very old and frail patients do not perform digital reporting independently, making caregiver- or professional-supported reporting a key component of coordinated telemonitoring workflows. This shifts remote monitoring from a purely digital interaction to a coordinated clinical process, where caregivers and nursing teams play a key operational role.

This shifts remote monitoring from a purely digital interaction to a coordinated clinical process, where caregivers and nursing teams play a key operational role.

Positioning telemonitoring as a flexible, caregiver-supported intervention ensures that frailty, cognitive impairment, or limited digital skills do not constitute exclusion criteria. Instead, these characteristics inform program design and organization, enabling telemonitoring to remain feasible, safe, and equitable across the full spectrum of frailty.

## The Frailty-Adaptive Remote Monitoring Framework

### Overview

The proposed framework defines 4 distinct frailty-based trajectories—robust, prefrail, frail, and palliative—each associated with specific monitoring objectives, workflow adaptations, and clinical responses.

**Figure 1.**
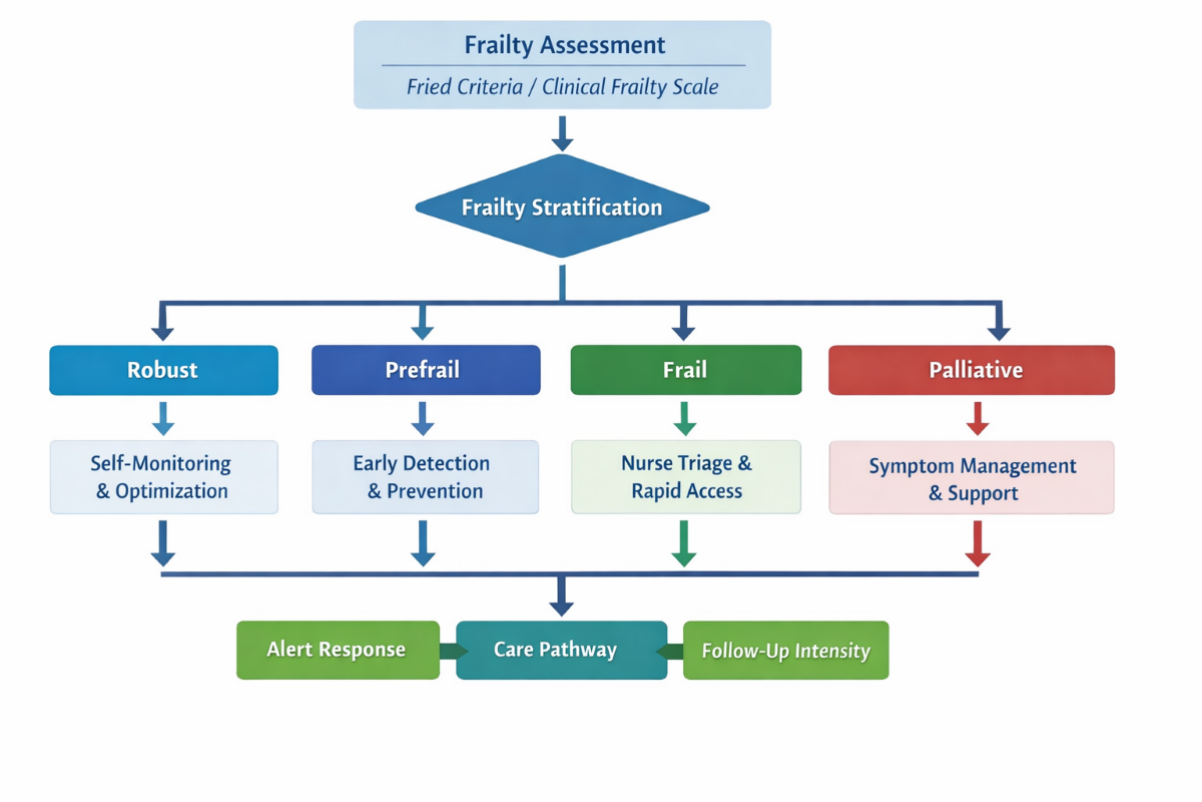
Frailty-adaptive telemonitoring framework for older adults with heart failure. This figure illustrates how remote monitoring strategies are adapted according to frailty status. After frailty assessment using validated tools (eg, Fried phenotype or Clinical Frailty Scale), patients are stratified into 4 trajectories: robust, prefrail, frail, and palliative. Each profile is associated with specific monitoring objectives, workflow adaptations, and response pathways, ranging from self-monitoring and optimization to nurse-led triage, rapid-access care, and symptom-guided support. Overall, the framework highlights the need for a stratified, patient-centered digital health approach.

Figure 1 provides a schematic overview of the frailty-adaptive telemonitoring framework, illustrating how patient stratification translates into differentiated monitoring strategies and care pathways.

Table 1 provides a structured overview of how remote-monitoring strategies can be aligned with frailty status to optimize clinical relevance, feasibility, and resource allocation.

**Table 1.** Frailty-based profiles and their adapted remote-monitoring strategies.

Frailty profile	Clinical characteristics	Primary monitoring objectives	Monitoring intensity & workflow adaptations	Typical interventions triggered
Robust	Preserved mobility, good cognition, low comorbidity burden	Optimization, empowerment, GDMT^a^ titration	Standard symptom questionnaire; patient self-reporting; minimal caregiver involvement	Diuretic adjustment, lifestyle advice, GDMT optimization
Prefrail	Early vulnerability, mild decline in gait, fatigue, early cognitive signs	Detection of early deterioration and reduction of risk of functional decline and hospitalization	Slightly increased monitoring frequency; simplified alerts; closer coordination with primary care and nurses	Early titration, nutritional support, mobility interventions, closer follow-up
Frail	Multimorbidity, polypharmacy, functional dependency, cognitive impairment	Crisis avoidance, stabilization, early identification of decompensation	Proxy reporting common (caregiver/nurse); structured triage; rapid-access day-hospital pathway; nurse-led adjustments	IV^b^ diuretics, iron repletion, geriatric assessment, medication adjustment
Palliative	Severe frailty, refractory symptoms, limited life expectancy, goal: comfort	Symptom-guided care, hospitalization avoidance, support at home	Highly simplified monitoring; caregiver-driven reporting; alerts focused on comfort/symptom distress rather than disease control	Symptom relief, comfort titration, avoiding ED^c^ visits, supporting home-based end-of-life care

a GDMT: guideline-directed medical therapy.

b IV: intravenous.

c ED: emergency department.

### Robust Older Adults: Optimization and Prevention

Robust older adults, characterized by preserved mobility, intact cognition, and low comorbidity burden, are the population most closely represented in early telemonitoring trials. In this group, remote monitoring primarily supports optimization of HF management, including early detection of congestion, reinforcement of adherence, lifestyle optimization, and guideline-directed medical therapy titration.

Evidence from trials such as Tele-HF, TIM-HF, TIM-HF2, and MORE-CARE, as well as meta-analyses, suggests that benefits in this population depend on program responsiveness and integration into multidisciplinary HF care [2,3,18,19].

Patients typically perform self-reporting independently, with high adherence and stable engagement. In this context, telemonitoring functions as a preventive strategy aimed at maintaining clinical and functional stability.

### Prefrail Patients: Early Detection and Functional Preservation

Prefrail individuals represent a transitional stage characterized by early vulnerability, including mild sarcopenia, reduced gait speed, fatigue, or early cognitive changes.

In this trajectory, the primary role of remote monitoring is the early detection of subtle clinical or functional deterioration. Changes in symptom patterns—such as increased fatigue, reduced activity tolerance, progressive dyspnea for similar exertion, or reduced ability to complete questionnaires independently—may signal early decompensation or loss of functional reserve. Increased caregiver involvement or delayed responses to alerts may also reflect declining autonomy.

Although direct evidence that telemonitoring prevents transition to frailty is lacking, early detection of clinical deterioration and timely intervention may contribute to preserving functional status and reducing hospitalization risk. This supports a model of slightly intensified monitoring, simplified alert systems, and closer coordination with primary care, nursing teams, and caregivers.

### Frail Patients: Stabilization and Crisis Avoidance

In frail older adults, HF is embedded within multimorbidity, polypharmacy, cognitive impairment, and functional dependency. These patients experience frequent exacerbations, high hospitalization rates, and reduced ability to engage independently in self-monitoring. Frailty has been consistently identified as a major predictor of hospitalization and mortality in populations with HF [11-13,17,20].

In this group, the effectiveness of telemonitoring depends less on patient digital capacity than on organizational structure. Proxy-assisted reporting by caregivers or health care professionals is common and enables continued participation despite functional limitations [4-6,21,22].

Remote monitoring therefore operates as a stabilization tool within an integrated care pathway. Key components include nurse-led triage, structured clinical verification of alerts, and rapid access to in-person reassessment. Typical interventions include intravenous diuretics, iron repletion, medication adjustment, and comprehensive geriatric assessment. Integrated cardiogeriatric models have demonstrated improved symptom control and reduced hospital utilization in this population [12,22,23].

The primary objective in this trajectory is crisis avoidance through early intervention and rapid care escalation rather than patient-driven self-management.

### Palliative Patients: Symptom Control and Home-Based Care

Patients in the palliative trajectory typically present with advanced HF, severe frailty, refractory symptoms, and limited life expectancy. Many express a preference to avoid hospitalization and remain at home. However, in practice, a large proportion still die in hospital, often following acute decompensation events [24,25].

When adapted to patient goals, remote monitoring may support symptom-guided care, early identification of distress, and avoidance of unnecessary emergency department visits. Monitoring strategies in this group are simplified and often rely on caregiver-driven reporting, with alert systems focused on symptom burden rather than disease optimization.

Evidence from palliative telehealth and home-based HF care suggests that such approaches can improve symptom control, reduce acute care use, and support home-based end-of-life care [26-28]. In this trajectory, remote monitoring shifts from disease management to a supportive role aimed at maintaining comfort and enabling care aligned with patient preferences.

These four trajectories illustrate how remote monitoring can be operationalized as a stratified, frailty-responsive care strategy rather than a uniform digital intervention.

## Clinical and Health System Implications

This viewpoint proposes a frailty-adaptive framework for remote monitoring in older adults with HF, structured around 4 clinical trajectories with differentiated monitoring objectives, workflows, and clinical responses. This framework provides an operational rather than purely conceptual approach by linking frailty profiles to concrete monitoring pathways, clinical responses, and organizational requirements. It challenges the prevailing model of uniform telemonitoring by positioning frailty as a primary determinant of digital health design rather than a secondary characteristic.

## Addressing Common Misconceptions and Barriers

A common misconception is that frail or palliative patients lack the capacity to engage with digital health interventions. In practice, remote monitoring in these populations rarely relies solely on patient-driven input. Data collection and alert responses can be supported by caregivers, home nurses, or health care professionals, and monitoring systems can be simplified or structured around nurse-led workflows. Multiple studies have shown that older adults—including those with frailty or mild cognitive impairment—can successfully participate in telemonitoring when programs are appropriately adapted [4-6,20-22].

Another concern relates to the potential burden on health care teams. However, evidence suggests that structured telemonitoring programs, particularly those integrating early detection and rapid intervention pathways, can reduce hospitalizations and emergency department visits, thereby decreasing overall health care use in high-risk HF populations [2,3,19,23,24,29].

Ethical concerns are also raised regarding the use of telemonitoring in palliative patients. However, studies in palliative telehealth demonstrate that remote monitoring can improve symptom control, reduce distressing hospital transfers, and support alignment with patient preferences, particularly when centered on comfort, shared decision-making, and caregiver support [26-28].

These considerations indicate that telemonitoring is not inherently unsuitable for frail or palliative populations; rather, its effectiveness depends on the extent to which programs are adapted to their specific needs and goals.

## Implications for Clinical Practice and Health Systems

Integrating frailty into the design of remote monitoring has direct implications for clinical programs and health systems. From a digital health perspective, this approach shifts telemonitoring from a uniform technological deployment to a stratified system design integrating patient-level heterogeneity. Telemonitoring strategies should be structured according to frailty profiles, with differentiated objectives, alert thresholds, follow-up intensity, and care pathways.

In practice, this requires tiered monitoring models, nurse-led triage, rapid-access pathways, and coordination with caregivers and community-based providers. At the system level, policy frameworks should support the integration of geriatric expertise into HF care, promote multidisciplinary coordination, and incentivize organizational models that link telemonitoring with rapid clinical response [22,23,30,31]. Digital health solutions should allow flexible configuration, including customizable alert thresholds, caregiver interfaces, and simplified reporting processes adapted to varying levels of functional and cognitive capacity.

## Digital Equity as a Core Dimension

Digital equity represents a central challenge in the implementation of remote monitoring in aging populations. Older adults frequently face structural barriers, including limited digital literacy, sensory impairments, cognitive decline, and variable access to caregivers. Without adaptation, these factors may lead to the exclusion of the most vulnerable patients from digital health interventions.

A frailty-adaptive approach addresses these challenges by embedding equity into program design. Key elements include simplified interfaces, proxy-supported data entry, educational support tailored to functional capacity, and monitoring workflows that do not rely exclusively on patient autonomy.

## Positioning Within Existing Literature

The effectiveness of telemonitoring in HF has been highly variable across studies, largely due to differences in program design, patient selection, and response workflows. By explicitly incorporating frailty into these dimensions, the proposed framework provides a potential explanation for part of this heterogeneity.

Variability in outcomes may reflect misalignment between monitoring strategies and patient vulnerability rather than limited efficacy. Frailty-based segmentation may therefore represent a key lever to improve both clinical impact and implementation success. This framework may also provide a basis for future risk stratification models and the integration of frailty into digital health algorithms.

Frailty should be considered not only a prognostic marker but a structuring principle for digital care pathways.

## Limitations

This viewpoint presents a conceptual framework rather than results from an interventional or comparative study. Although grounded in published evidence and real-world clinical experience, the proposed model has not been empirically validated as a unified strategy.

Some elements are derived from a French telemonitoring program implemented within the national ETAPES framework; however, the principles described—including frailty segmentation, proxy-supported reporting, and nurse-led triage—are intended to be generalizable across different health care systems and digital infrastructures.

Future research should prospectively evaluate this framework, including stratified outcomes according to frailty level and its impact on hospitalization, quality of life, and health care utilization.

## Toward Frailty-Adaptive Digital HF Care

Remote monitoring should not remain a uniform technological intervention applied identically across a heterogeneous aging population. Its value depends on its ability to adapt both to frailty status and to the dynamic progression of HF. Robust patients may benefit primarily through optimization and empowerment; prefrail patients through early detection and timely intervention; frail patients through crisis avoidance and structured rapid-access care; and palliative patients through comfort-focused support, stabilization, and the preservation of home-based dignity.

A frailty-adaptive remote monitoring strategy is also essential for digital health equity, ensuring that older adults with the greatest vulnerabilities are not excluded from digital transformation but are meaningfully included within it.

A frailty-based conceptual model represents a necessary evolution in digital HF care and provides a framework for more meaningful and sustainable telemonitoring programs. One-size-fits-all telemonitoring is fundamentally misaligned with the biology and care trajectories of older adults.

## Abbreviations

ETAPES: Expérimentations de Télémédecine pour l’Amélioration des Parcours en Santé
HF: heart failure
Tele-HF: Telemonitoring to Improve Heart Failure Outcomes
TIM-HF: Telemedical Interventional Monitoring in Heart Failure

## References

[1] Chaudhry SI, Mattera JA, Curtis JP (2010). Telemonitoring in patients with heart failure. N Engl J Med.

[2] Koehler F, Koehler K, Deckwart O (2018). Efficacy of telemedical interventional management in patients with heart failure (TIM-HF2): a randomised, controlled, parallel-group, unmasked trial. Lancet.

[3] Girerd N, Barbet V, Seronde MF (2025). Association of a remote monitoring programme with all-cause mortality and hospitalizations in patients with heart failure: national-scale, real-world evidence from a 3-year propensity score analysis of the TELESAT-HF study. Eur J Heart Fail.

[4] Cajita MI, Gleason KT, Han HR (2016). A systematic review of mHealth-based heart failure interventions. J Cardiovasc Nurs.

[5] Kruse C, Fohn J, Wilson N, Nunez Patlan E, Zipp S, Mileski M (2020). Utilization barriers and medical outcomes commensurate with the use of telehealth among older adults: systematic review. JMIR Med Inform.

[6] Lam K, Lu AD, Shi Y, Covinsky KE (2020). Assessing telemedicine unreadiness among older adults in the United States during the COVID-19 pandemic. JAMA Intern Med.

[7] Fried LP, Tangen CM, Walston J (2001). Frailty in older adults: evidence for a phenotype. J Gerontol A Biol Sci Med Sci.

[8] Rockwood K, Mitnitski A (2007). Frailty in relation to the accumulation of deficits. J Gerontol A Biol Sci Med Sci.

[9] Clegg A, Young J, Iliffe S, Rikkert MO, Rockwood K (2013). Frailty in elderly people. Lancet.

[10] Afilalo J, Alexander KP, Mack MJ (2014). Frailty assessment in the cardiovascular care of older adults. J Am Coll Cardiol.

[11] McNallan SM, Chamberlain AM, Gerber Y (2013). Measuring frailty in heart failure: a community perspective. Am Heart J.

[12] Vidán MT, Blaya-Novakova V, Sánchez E, Ortiz J, Serra-Rexach JA, Bueno H (2016). Prevalence and prognostic impact of frailty and its components in non-dependent elderly patients with heart failure. Eur J Heart Fail.

[13] Denfeld QE, Winters-Stone K, Mudd JO, Gelow JM, Kurdi S, Lee CS (2017). The prevalence of frailty in heart failure: a systematic review and meta-analysis. Int J Cardiol.

[14] Savarese G, Lund LH (2017). Global public health burden of heart failure. Card Fail Rev.

[15] Rodríguez-Pascual C, Paredes-Galán E, Ferrero-Martínez AI (2017). The frailty syndrome is associated with adverse health outcomes in very old patients with stable heart failure: a prospective study in six Spanish hospitals. Int J Cardiol.

[16] Uchmanowicz I, Gobbens RJJ (2015). The relationship between frailty, anxiety and depression, and health-related quality of life in elderly patients with heart failure. Clin Interv Aging.

[17] Pandey A, Kitzman D, Whellan DJ (2019). Frailty among older decompensated heart failure patients: prevalence, association with patient-centered outcomes, and efficient detection methods. JACC Heart Fail.

[18] Boriani G, Da Costa A, Quesada A (2017). Effects of remote monitoring on clinical outcomes and use of healthcare resources in heart failure patients with biventricular defibrillators: results of the MORE-CARE multicentre randomized controlled trial. Eur J Heart Fail.

[19] Inglis SC, Clark RA, Dierckx R, Prieto-Merino D, Cleland JGF (2015). Structured telephone support or non-invasive telemonitoring for patients with heart failure. Cochrane Database Syst Rev.

[20] Afilalo J, Karunananthan S, Eisenberg MJ, Alexander KP, Bergman H (2009). Role of frailty in patients with cardiovascular disease. Am J Cardiol.

[21] Eberly LA, Kallan MJ, Julien HM (2020). Patient characteristics associated with telemedicine access for primary and specialty ambulatory care during the COVID-19 pandemic. JAMA Netw Open.

[22] Kvedar JC, Fogel AL, Elenko E, Zohar D (2016). Digital medicine’s march on chronic disease. Nat Biotechnol.

[23] Whellan DJ, Goodlin SJ, Dickinson MG (2014). End-of-life care in patients with heart failure. J Card Fail.

[24] Warraich HJ, Yancy CW (2019). Failure is not an option: optimizing care for heart failure patients at the end of life. JACC Heart Fail.

[25] Gadoud A, Jenkins SMM, Hogg KJ (2013). Palliative care for people with heart failure: summary of current evidence and future direction. Palliat Med.

[26] Bakitas MA, Dionne-Odom JN, Ejem DB (2020). Effect of an early palliative care telehealth intervention vs usual care on patients with heart failure: the ENABLE CHF-PC randomized clinical trial. JAMA Intern Med.

[27] Calton B, Shibley WP, Cohen E (2020). Patient and caregiver experience with outpatient palliative care telemedicine visits. Palliat Med Rep.

[28] Gelfand SL, Lakin JR, Sciacca KR (2022). Specialty-aligned palliative care: responding to the needs of a tertiary care health system. J Pain Symptom Manage.

[29] Koehler F, Winkler S, Schieber M (2012). Telemedicine in heart failure: pre-specified and exploratory subgroup analyses from the TIM-HF trial. Int J Cardiol.

[30] Naylor MD, Brooten DA, Campbell RL, Maislin G, McCauley KM, Schwartz JS (2004). Transitional care of older adults hospitalized with heart failure: a randomized, controlled trial. J Am Geriatr Soc.

[31] Boyd CM, Landefeld CS, Counsell SR (2008). Recovery of activities of daily living in older adults after hospitalization for acute medical illness. J Am Geriatr Soc.

